# Effect of Discectomy on Dynesys Dynamic Fixation in the Treatment of Lumbar Degenerative Diseases

**DOI:** 10.1155/2021/3043645

**Published:** 2021-12-30

**Authors:** Chen Zhao, Liehua Liu, Lei Luo, Pei Li, Yiyang Wang, Lichuan Liang, Xueping Wen, Dianming Jiang, Qiang Zhou

**Affiliations:** Department of Orthopedics, The Third Affiliated Hospital of Chongqing Medical University, Chongqing, China

## Abstract

**Objective:**

To compare the effect of decompression of the spinal canal with or without discectomy on the clinical efficacy of Dynesys dynamic fixation treatment in lumbar degenerative diseases.

**Methods:**

A total of 62 patients treated for single-segment lumbar degenerative disease from October 2010 to November 2017 were retrospectively analyzed. All patients underwent decompression of the spinal canal with Dynesys dynamic fixation and were divided into two groups. Twenty-seven patients in group A did not undergo discectomy, and 35 patients in group B underwent discectomy. The intervertebral height, range of motion, Pfirrmann grade of the surgical segment and the upper adjacent segment, function scores, and operation information were compared.

**Results:**

All patients were followed up for an average of 30.7 ± 11.5 months. At the final follow-up, the intervertebral height and range of motion of the surgical segment decreased significantly in both group A and B (*p* < 0.05), the range of motion of the upper adjacent segment increased significantly (*p* < 0.05), and the intervertebral height did not change significantly (*p* < 0.05). The retained percentages of surgical segment intervertebral height and ROM in group A were significantly better than those in group B (*p* < 0.05). The intervertebral height (*p* > 0.05) and range of motion (*p* < 0.05) of the surgical segment in group A were higher than those in group B. The surgical segment Pfirrmann grading of group A was better than that of group B (*p* < 0.05).

**Conclusion:**

Dynesys in the treatment of lumbar degenerative diseases may lead to a good clinical effect. In selected cases without discectomy, the range of motion and intervertebral height may be better preserved, and disc degeneration may be reduced.

## 1. Introduction

Lumbar degenerative diseases are among of the common diseases in spinal surgery, including disc herniation, spondylolisthesis, and spinal stenosis. Although conservative treatment may be effective, there are still some patients who require surgical treatment due to severe symptoms or ineffective conservative treatment [1]. Lumbar spinal fusion is a well-utilized surgical method in the treatment of lumbar degenerative diseases that can effectively relieve nerve compression, stabilize the spine, and achieve good clinical effects [2]. However, some complications can develop during follow-up, such as adjacent segment disease (ASD), bone nonunion, and pseudarthrosis [3].

Lumbar dynamic fixation is a technique that has been used in the treatment of lumbar degenerative diseases in recent years to avoid complications due to lumbar fusion. At present, the Dynesys system is most widely used in all dynamic fixed systems [4–6]. The Dynesys system consists of pedicle screws and elastic synthetic compounds [7], which can stabilize the spine while retaining the mobility of surgical segments and reduce the incidence of ASD. However, the clinical efficacy of this technique is still controversial [8–10].

The surgical procedure mainly included nerve decompression and dynamic fixation implantation. Discectomy during nerve decompression may affect the height and range of motion of the intervertebral space, so discectomy performed during the operation may affect the clinical outcome. However, there are very few reports. To explore the factors affecting the clinical efficacy of dynamic fixation, we retrospectively analyzed the outcomes of 62 patients with single-segment lumbar degenerative disease to observe the effect of decompression of the spinal canal with or without discectomy in terms of the clinical efficacy of Dynesys dynamic fixation for the treatment of lumbar degenerative disease.

## 2. Materials and Methods

### 2.1. The General Data

Inclusion criteria: (1) patients who were diagnosed with single-segment lumbar degenerative diseases, including disc herniation, spinal stenosis, and lumbar degenerative spondylolisthesis were included; (2) patients exhibited no significant decrease in the intervertebral height and were not diagnosed with kyphosis; (3) the patient had undergone spinal decompression and Dynesys dynamic fixation; and (4) the age of patients was ≥18 years.

Exclusion criteria: (1) patients who had undergone spinal surgery in any segment; (2) their spinal condition could not be combined with severe osteoporosis; and (3) patients could also not exhibit diseases that may have affected clinical observation, such as cervical spondylosis and thoracic stenosis.

Patients treated for single-segment lumbar degenerative disease from October 2010 to November 2017 were retrospectively analyzed. This study was approved by the hospital ethics committee (IRB No. 2012014). A total of 62 patients were included in the study for statistical analysis and were divided into two groups according to whether they underwent discectomy during the operation. All the operations were performed by the same surgeon. There were 27 patients in group A (without discectomy), including 9 males and 18 females, with an average age of 49.0 ± 13.8 years. There were 19 patients being treated for L4-5 segment and 8 patients with L5-S1 segment. Also, there were 9 cases of disc herniation, 7 cases of degenerative spondylolisthesis, and 11 cases of spinal stenosis. Group B consisted of 35 patients with discectomy, including 15 males and 20 females, with an average age of 44.4 ± 10.3 years, 20 patients with L4-5 segment, and 15 patients with L5-S1 segment. Also, there were 5 cases of disc herniation, 16 cases of degenerative spondylolisthesis, and 14 cases of spinal stenosis. There were no statistically significant differences in general data between the two groups (*p* > 0.05).

### 2.2. Preoperative Preparation

All patients underwent 3–6 months of conservative treatment before surgery, and surgical treatment was considered when improvement was poor. After admission, patients underwent routine blood tests, liver and kidney function tests, blood coagulation tests, electrocardiograms, and chest radiographs. X-ray (anteroposterior, lateral, extension, and flexion), computerized tomography (CT), and magnetic resonance imaging (MRI) were performed in all patients to determine the range of motion (ROM) of the lumbar spine and disc herniation. Patients were instructed to perform functional exercises, such as lung function exercise and lower limb functional exercise, for quicker postoperative recovery.

### 2.3. Surgical Technique

Patients received general anesthesia and then were placed in the prone position with appropriate hip and knee flexion to perform lumbar spinal flexion. A posterior midline incision was made according to the surgical segment. The paraspinal muscle was dissected along the paraspinous process to expose the interlaminar foramen. The inferior facet, lamina, and superior facet were partially excised; the lateral recess was fully enlarged, the ligamentum flavum was removed, and the nerve roots and intervertebral discs were exposed. If the patient has bilateral symptoms, bilateral decompression is performed. Discectomy was not performed if the spinal dura mater pulsated well, nerve roots were relaxed, and the annulus fibrosus was not damaged (group A). Otherwise, the spinal dura mater and nerve root were pulled slightly to the medial side, then the annulus fibrosus was excised appropriately, and extruded disc fragments and tender disc tissues were removed (group B). The Wiltse approach was used to expose the bilateral pedicle screw entry points in the same incision, and the Dynesys pedicle screws were implanted in the bilateral vertebral pedicle. The operating table was adjusted to restore the lumbar spine to a neutral position, and then, the distance was measured between the screws on both sides and spacers were installed according to the technical requirements. In the case of lumbar spondylolisthesis, the spacing distance should not be too large, and the tightening force of the rope should be greater. Drainage tubes were placed on both sides, and the wound was sutured after flushing.

### 2.4. Postoperative Management

Postoperative vital signs of the patients were observed, and infection prevention, anti-inflammatory, dehydration, and neuronutritional treatment were given. Two days after the operation, patients could wear a waist supporter and ambulate gradually. Drainage tubes were removed when the drainage rate was less than 30 ml/d. Patients were followed up postoperatively at 1, 3, 6, 12, 18, and 24 months and then once a year after 2 years. X-rays were performed at each follow-up, and an MRI was performed at 2 and 4 years after operation.

### 2.5. Statistical Analysis

Clinical outcomes and imaging changes were recorded in all patients. Lumbar intervertebral height was assessed by lateral X-rays, and ROM was assessed by extension and flexion X-rays. The intervertebral height was calculated by the mean of the leading-edge height and the trailing-edge height. ROM was measured by the angle between the upper and lower endplates, and the ROM was the difference between the angle of extension and flexion. The degree of intervertebral disc degeneration was evaluated by Pfirrmann grading [11].

### 2.6. The Data in This Study Were Statistically Analyzed Using SPSS 19.0

The operative time, blood loss, visual analog scale (VAS), Oswestry disability index (ODI), intervertebral height, ROM, retained percentage of intervertebral height, and ROM between the two groups were analyzed by *t*-tests. Intervertebral height and ROM in each group between preoperative and final follow-up were analyzed by paired *t*-tests. VAS and ODI in each group at preoperative, postoperative, and final follow-up were analyzed by repeated ANOVA. The Pfirrmann grading of intervertebral discs and complications were analyzed by the chi-square test.

## 3. Results

All patients underwent operation successfully without serious complications. The mean operation times of group A and group B were 194.4 ± 42.5 min and 185.3 ± 31.3 min, respectively. The average blood loss in groups A and B was 307.4 ± 199.9 ml and 297.7 ± 188.2 ml, respectively. There was no significant difference in operation time or blood loss between the two groups (*p* > 0.05).

All patients were followed up with an average of 30.7 ± 11.5 m, among which group A was followed up with an average of 29.4 ± 9.0 m and group B with an average of 31.6 ± 13.1 m. There was no significant difference between the two groups (*p* > 0.05). VAS and ODI of group A and B are shown in Table 1. The VAS scores of low back pain and leg pain in both groups were significantly improved after surgery (*p* < 0.05), the VAS of low back pain in the final follow-up was significantly improved compared with that after surgery (*p* < 0.05), and the VAS of leg pain was improved, but there was no significant difference (*p* > 0.05). The ODI scores in both groups were significantly improved after surgery (*p* < 0.05) and improved further at the final follow-up (*p* < 0.05). There was no significant difference in VAS and ODI between the two groups at each time point (*p* > 0.05).

The intervertebral height and the ROM of groups A and B are shown in Table 2. There was no significant difference in the intervertebral height and ROM of the surgical segment and the upper adjacent segment between group A and group B before surgery (*p* > 0.05). At the final follow-up, the intervertebral height and ROM of the surgical segment decreased significantly in both groups (*p* < 0.05). The intervertebral height of the surgical segment at the final follow-up in group A was higher than that in group B (*p* > 0.05), and the ROM was significantly higher than that of group B (*p* < 0.05). The retained percentages of surgical segment intervertebral height and ROM in group A were significantly better than those in group B (*p* < 0.05). There was no significant change in the intervertebral height of the upper adjacent segment in groups A and B (*p* < 0.05), the ROM increased significantly (*p* < 0.05), and there was no significant difference between the two groups (*p* > 0.05).

**Figure 1:**
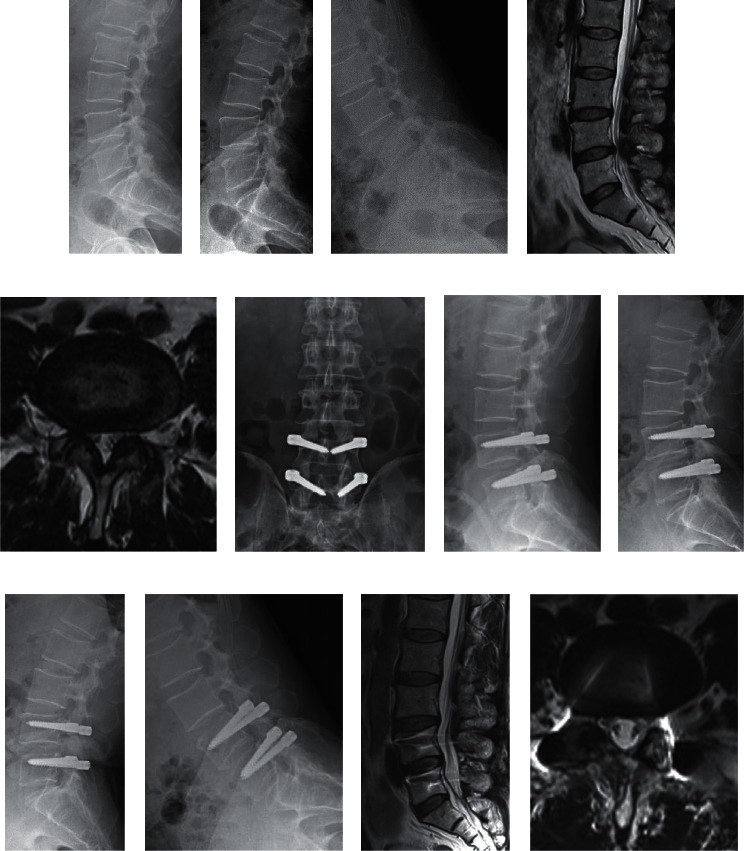
The typical cases in group A. A 57-year-old male patient developed L4/5 spinal stenosis who was assigned group A. A–E show the preoperative X-ray and MRI scans. The intervertebral height and ROM of surgical were 11.35 mm and 5.5°, respectively. MRI scans showed the spinal stenosis was severe on the right side. The Pfirrmann grading was grade 3. The patient underwent spinal decompression without discectomy and Dynesys dynamic fixation. F and G show the 1-week postoperative X-rays. H–L show the 2-year postoperative X-ray and MRI scans. At the 2-year follow-up, the intervertebral height and ROM of surgical were 10.5 mm and 4.4°, and the retained percentages were 92.5% and 80.0%, respectively. MRI scans showed there was no stenosis in the spinal canal and disc herniation improved. The Pfirrmann grading was grade 2.

The Pfirrmann grading changes in the surgical segment and the upper adjacent segment in groups A and B are shown in Table 3. There was no significant difference between the two groups in the preoperative Pfirrmann grading of the surgical segment and the upper adjacent segment (*p* > 0.05). At the final follow-up, 22.2% of patients in group A showed Pfirrmann grading improvement, 51.9% were unchanged, and 25.9% degenerated. In group B, 8.6% of patients had improved, 31.4% were unchanged, and 60.0% degenerated. There was a significant difference in surgical segment Pfirrmann grading in each group compared with preoperatively (*p* < 0.05). At the final follow-up, the Pfirrmann grading of group A was better than that of group B (*p* < 0.05). At the final follow-up, the Pfirrmann grade of the upper adjacent segment in group A was unchanged in 92.6% of cases and degenerated in 7.4%. In group B, the grade was unchanged in 88.6% of cases and degenerated in 11.4%. There was no significant difference in upper adjacent segment Pfirrmann grading in each group compared with preoperative scores (*p* > 0.05), and there was no significant difference between the two groups (*p* > 0.05). The typical cases in group A are shown in Figure 1.

One patient in group A had poor wound healing. One patient in group B had cerebrospinal fluid leakage, and another patient had upper adjacent segmental instability at 4 years after surgery. Patients with adjacent segmental instability exhibited no clinical symptoms and did not receive special treatment. The other patients were cured after conservative treatment. The incidence of complications in group A and B was 3.7% and 5.7%, respectively. There was no significant difference in the incidence of complications between the two groups (*p* > 0.05).

## 4. Discussion

One of the main purposes of surgical treatment for lumbar degenerative diseases is to relieve nerve symptoms such as lower limb pain and numbness, so most surgical procedures need nerve decompression. During lumbar degeneration, nerve compression could be caused by hyperplasia of facets, thickened ligamentum flavum, and herniated discs. Therefore, nerve decompression involves partial lamina, facet, and ligamentum flavum removal, combined with or without discectomy. In our experience, the operation can be performed without discectomy when the annulus fibrosus is not fissured, and adequate nerve decompression can be obtained by excision of the lamina, facet, and ligamentum flavum. The results of this study also confirm that patients with or without discectomy may have the same improved clinical effects in lower back pain and leg pain under appropriate circumstances. In addition, we recommend decompression through the interlaminar approach and preserving facet joints as much as possible to better preserve spinal stability.

The Dynesys system functions through spacers and connectors. The spacers limit lumbar hyperextension, while connectors limit hyperflexion, stabilizing the surgical segment while retaining ROM. A meta-analysis reported that, after Dynesys fixation, the mean ROM of the surgical segment decreased from 6.64° to 3.64°, retaining 54.8% [12]. Dynesys is a pedicle dynamic system, and spacers are implanted between the ends of the screws. Therefore, this system provides stronger support in the posterior column compared to the anterior column, while the anterior column's main support structure remains the intervertebral disc. Discectomy may affect the height of the intervertebral space at the surgical segment, thus affecting the range of motion. The results of this study showed that the retained height of the intervertebral space was 94.5 ± 12.0% and 79.6 ± 20.0% in groups A and B postoperatively, and the retained ROM was 79.6 ± 19.4% and 54.0 ± 25.5%, respectively. The retention ROM and intervertebral space height in group A were significantly higher than those in group B, indicating that the Dynesys dynamic fixation system could better maintain the height of the intervertebral space and retain the ROM when the operation was performed without discectomy.

The intervertebral disc is an important tissue for load transmission. Studies have shown that high load compressive stress accelerates the degeneration of intervertebral discs, while low load compressive stress can promote the regeneration of intervertebral disc nucleus pulposus cells [13]. Dynesys dynamic fixation can reduce the load on the facet joint and relieve disc pressure, while retaining the low load dynamic compressive stress on the disc. Vaga et al. observed changes in intervertebral discs after Dynesys fixation by MRI and found that 61% of the surgical segments had an increase in glycosaminoglycans (GAG) [14]. Yilmaz et al. reported that Pfirrmann grading improved in 34% of patients after dynamic fixation, worsened in 13.5%, and remained unchanged in 52.5% [15]. In this study, Pfirrmann grading improved in 22.2% of patients in group A and worsened in 25.9%, while it improved in 8.6% of patients in group B and worsened in 60.0%. There was no disc improvement in Pfirrmann grading in adjacent segments. Therefore, we believe that Dynesys dynamic fixation may slow down the degeneration of intervertebral discs and even promote regeneration. However, if discectomy damaged the annulus fibrosus and nucleus pulposus, then disc degeneration accelerated and regeneration decreased.

The effect of dynamic fixation on adjacent segments has been controversial. Reportedly, 7% of patients developed ASD after Dynesys system fixation at a mean follow-up of 33.5 months [16], while 1.9% to 30.3% of patients developed ASD after lumbar fusion at the 5-year follow-up [17]. Adjacent disc degeneration is a natural physiological phenomenon, but it may be accelerated as the ROM of adjacent segments increases. One study reported that the mean increases in ROM of adjacent segments after Dynesys fixation and posterior lumbar interbody fusion were 0.33° and 1.15°, respectively [12]. The results of this study showed that the ROM of the upper adjacent segments in group A and group B significantly increased, by 1.6° and 1.8°, respectively, compared with that before the operation. The Pfirrmann grading of upper adjacent segments in group A worsened in 7.4%, while in group B, the percentage that worsened was 11.4%. In cases without discectomy, the ROM of the surgical segment may be better preserved, while the ROM of adjacent segments that needs to compensate is smaller, so the risk factors for the degeneration of adjacent segments are reduced. In this study, the increase in the ROM of adjacent segments and the aggravation of disc degeneration in group A were less than those in group B, but there was no significant difference between the two groups. Further studies are needed to prove whether decompression without discectomy can more effectively prevent the degeneration of adjacent segments.

This study also has some defects, such as small number of cases were studied, cases were not of a single disease, and the follow-up time was short. The results of this study need to be further confirmed by more studies.

## 5. Conclusions

We believe that spinal decompression combined with Dynesys dynamic fixation can achieve good clinical efficacy in the treatment of lumbar degenerative diseases. In selected cases, performing surgery without discectomy can better preserve the ROM and intervertebral height and reduce disc degeneration.

## Figures and Tables

**Table 1:** Clinical outcomes.

Group	*N*	Low back pain			Leg pain			OID (%)		
		Pre	Post	FFU	Pre	Post	FFU	Pre	Post	FFU
A	27	3.9 ± 1.3	1.9 ± 0.6^*∗*^	0.9 ± 0.7^‡^	5.0 ± 1.0	1.3 ± 0.8^†^	0.9 ± 0.7	42.4 ± 14.7	16.7 ± 5.6^§^	4.8 ± 3.4^II^
B	35	4.2 ± 1.2	2.0 ± 0.7^*∗*^	0.9 ± 0.6^‡^	4.9 ± 0.9	1.2 ± 0.8^†^	0.9 ± 0.8	48.1 ± 14.8	15.1 ± 5.3^§^	5.9 ± 3.0^II^
t		−0.76	−0.68	−0.18	0.62	0.48	−0.03	−1.51	1.22	−1.38
p		0.45	0.50	0.86	0.54	0.64	0.98	0.14	0.23	0.17

Pre: preoperative; Post: postoperative; FFU: final follow-up. ^*∗*^^†^Repeated ANOVA results showed the VAS scores of low back pain and leg pain in the two groups were statistically different between preoperation and postoperation (*P* < 0.05). ^‡^Repeated ANOVA results showed the VAS scores of low back pain in the two groups were statistically different between postoperation and the final follow-up (*P* < 0.05). ^§II^Repeated ANOVA results showed the ODI in the two groups was statistically different between preoperation and postoperation and postoperation and the final follow-up (*P* < 0.05).

**Table 2:** Radiography outcomes.

Group	*n*	S-intervertebral height (mm)			S-ROM (°)			U-intervertebral height (mm)		U-ROM (°)	
		Pre	FFU	R (%)	Pre	FFU	R (%)	Pre	FFU	Pre	FFU
A	27	9.7 ± 1.9	9.1 ± 2.0^*∗*^	94.5 ± 12.0	6.7 ± 2.7	5.2 ± 1.9^†^	79.6 ± 19.4	11.2 ± 1.4	10.9 ± 1.5	6.0 ± 2.4	7.6 ± 3.2^§^
B	35	10.2 ± 2.0	8.0 ± 2.4^*∗*^	79.6 ± 20.0	6.1 ± 2.5	3.2 ± 1.8^†^	54.0 ± 25.5	11.6 ± 2.3	11.5 ± 1.6	6.6 ± 2.6	8.4 ± 3.9^§^
t		−0.95	1.92	3.44	0.96	4.05	4.34	−0.85	−1.50	−0.88	−0.80
P		0.35	0.06	0.00	0.34	0.00	0.00	0.40	0.14	0.38	0.43

Pre: preoperative; FFU: final follow-up; R: retention percentage. S-intervertebral height: surgical segment intervertebral height; S-ROM: surgical segment range of motion. U-intervertebral height: upper adjacent segment intervertebral height; U-ROM: upper adjacent segment range of motion. ^*∗*^*T*-test results showed the surgical segment intervertebral height in the two groups was statistically different between preoperation and the final follow-up (*p* < 0.05). ^†^*T*-test results showed the surgical segment range of motion in the two groups was statistically different between preoperation and the final follow-up (*p* < 0.05). ^§^*T*-test results showed the upper adjacent segment range of motion in the two groups was statistically different between preoperation and the final follow-up (*p* < 0.05).

**Table 3:** Pfirrmann grading.

Pre	FFU of group A (*n*)				FFU of group B (*n*)			
	2	3	4	5	2	3	4	5
2	3/11^*∗*^	0/2^*∗*^			0/14^*∗*^	1/1^*∗*^	1/0^*∗*^	
3	5/12^*∗*^	7/1^*∗*^	4/0^*∗*^		1/0^*∗*^	5/17^*∗*^	7/3^*∗*^	5/0^*∗*^
4		1/0^*∗*^	4/1^*∗*^	3/0^*∗*^		2/0^*∗*^	6/0^*∗*^	7/0^*∗*^

Pre: preoperative; FFU: final follow-up. ^*∗*^Upper adjacent segment data.

## Data Availability

The datasets used during the current study are available from the corresponding author on reasonable request.
